# Exploiting Metabolic Vulnerabilities in Acute Myeloid Leukemia: Rationale and Evidence for Combining Metabolic Modulators with Conventional Chemotherapy

**DOI:** 10.3390/ph19091384

**Published:** 2026-09-01

**Authors:** Usman Ali Shams, Fawad Inayat, Muhammad Asif Zeb, Maryam, Sulaiman Shams, Muhammad Jawad Ullah, Silvia Jiménez-Morales

**Affiliations:** 1 Department of Hematology, University of Health Sciences, Lahore 54000, Pakistan; 2 Department of Biochemistry, Abdul Wali Khan University, Mardan 23200, Pakistan; fawad.313b@gmail.com (F.I.); sulaiman@awkum.edu.pk (S.S.); 3 Institute of Paramedical Sciences, Khyber Medical University, Peshawar 25000, Pakistan; muhammadasif.ipms@kmu.edu.pk; 4 Department of Chemistry, Islamia College University, Peshawar 25000, Pakistan; maryamashrafshah98@gmail.com; 5 Department of Allied Health Sciences, Iqra National University, Peshawar 25000, Pakistan; jawadkhalil3132@gmail.com; 6 Laboratorio de Innovación y Medicina de Precisión, Núcleo A, Instituto Nacional de Medicina Genómica, Mexico City 14610, Mexico

**Keywords:** acute myeloid leukemia, metabolic reprogramming, glycolysis, oxidative phosphorylation, glutamine metabolism, chemotherapy, drug combination, targeted therapy

## Abstract

Acute myeloid leukemia (AML) represents a heterogeneous group of hematological malignancies characterized by uncontrolled proliferation of myeloid progenitors and accumulation of immature blasts in the bone marrow. Metabolic reprogramming is now recognized as a core hallmark of AML, generating dependencies that distinguish leukemic cells from normal hematopoietic stem and progenitor cells and that can be exploited therapeutically. In this review we follow a single connected line of argument: we first place metabolic rewiring within the broader hallmarks of cancer, then describe the principal metabolic programs altered in AML and the specific features that distinguish AML from other malignancies. We next examine the inhibitors and drugs that target each of these pathways, linking every drug class to its mechanism of synergy with chemotherapy, the preclinical and clinical evidence available, and its association with outcome in AML. We then consider multi-target (combination) therapy as a distinct opportunity, and finally the principal challenges that remainsafety and tolerability, the metabolic heterogeneity and plasticity of AML, and the design of biomarker-guided trials. Multiple classes of metabolic drugs are discussed, including glycolysis inhibitors, oxidative phosphorylation inhibitors, glutamine metabolism antagonists, fatty acid oxidation modulators, and redox-active compounds. Despite significant challenges, targeting cellular metabolism represents a promising strategy to enhance therapeutic outcomes in patients with AML.

## 1. Introduction

Acute myeloid leukemia (AML) is a devastating hematological malignancy representing a major proportion of adult acute leukemias, with recent epidemiological estimates indicating over 20,000 new cases annually in the United States [1]. Globally, AML affects approximately 850,000 individuals, contributing to around 420,000 deaths each year. Despite advances in intensive chemotherapy protocols and the introduction of targeted therapies such as the anti-CD33 antibody–drug conjugate gemtuzumab ozogamicin and isocitrate dehydrogenase (IDH) inhibitors for patients with IDH-mutant AML, the prognosis remains poor for many patients, particularly those with refractory or relapsed disease [2].

To place the metabolic changes discussed in this review in context, it is useful to recall that altered energy metabolism is one of the established hallmarks of cancer. Proliferating tumor cells reprogram their metabolism to take up large amounts of glucose and glutamine, to generate biosynthetic precursors and reducing equivalents, and to maintain redox balance, in addition to producing ATP. These features are shared by many cancers and by rapidly proliferating normal cells; what matters therapeutically is not the presence of a given pathway but the degree to which leukemic cells become dependent on it. Throughout this review we therefore emphasize, wherever the evidence allows, the metabolic features that are comparatively specific to AML rather than common to all malignancies.

Emerging evidence indicates that AML is characterized by extensive metabolic rewiring that distinguishes leukemic cells from normal hematopoietic stem and progenitor cells (HSPCs) [3]. These metabolic alterations arise from complex interactions between genetic mutations, epigenetic modifications, and microenvironmental factors, resulting in distinct pathway activities and metabolic fluxes [4]. Increased throughput in glycolysis, glucose oxidation, serine and one-carbon metabolism, fatty acid metabolism, and the tricarboxylic acid (TCA) cycle, coupled with reduced urea cycle activity, collectively define the metabolic landscape of AML [5]. Targeting these metabolic dependencies has emerged as an appealing strategy to enhance the efficacy of standard chemotherapeutic agents, particularly cytarabine and anthracyclines such as idarubicin [6]. Metabolic inhibitors are postulated to amplify stress signals induced by chemotherapy-related DNA damage or to restore chemosensitivity through direct effects on drug targets [7]. Several metabolic agents are currently undergoing preclinical evaluation and clinical trials in combination with conventional chemotherapy for advanced-stage AML [8]. Furthermore, recent advances in single-cell analysis and hybrid droplet mass spectrometry for measuring metabolic fluxes have opened new avenues for characterizing targetable metabolic vulnerabilities and facilitating patient stratification [9].

While several reviews have cataloged the metabolic landscape of hematological malignancies, this review provides a dedicated conceptual framework focused specifically on the mechanistically defined synergy between metabolic modulators and conventional or targeted agents in AML. Organized as a single connected narrative, we first describe the principal metabolic programs reprogrammed in AML and evaluate the inhibitors targeting each pathway. We link these combinations directly to their mechanisms of synergy categorized into distinct functional nodes: DNA damage response amplification, redox sensitization, mitochondrial priming, and stem-cell eradication and examine their associations with clinical outcomes. We subsequently discuss multi-target combination therapy as a dedicated opportunity and address critical translational challenges, separating actionable driver-mutation biomarkers from descriptive metabolic profiles to outline a pragmatic roadmap for biomarker-guided combination trials before concluding.

### Literature Search Strategy and Selection Criteria

A comprehensive literature search was conducted across PubMed, Google Scholar, Web of Science to identify relevant studies published between January 2015 and July 2026, with particular emphasis on recent literature from 2023 to 2026. Search strings included combinations of the terms: ‘acute myeloid leukemia’, ‘AML’, ‘metabolic reprogramming’, ‘glycolysis’, ‘oxidative phosphorylation’, ‘glutaminolysis’, ‘fatty acid oxidation’, ‘chemotherapy synergy’, ‘venetoclax’, and specific drug names (e.g., ‘CB-839’, ‘IACS-010759’, ‘AZD3965’, ‘2-deoxyglucose’). Articles were selected based on their relevance to metabolic vulnerabilities, combination therapies, and clinical trials in AML. Peer-reviewed original research, mechanistic preclinical studies, and clinical trial reports were prioritized. Non-English articles, studies lacking direct translational relevance to AML, and abstracts without complete dataset reporting were excluded.

## 2. Metabolic Reprogramming in Acute Myeloid Leukemia

**Figure 1:**
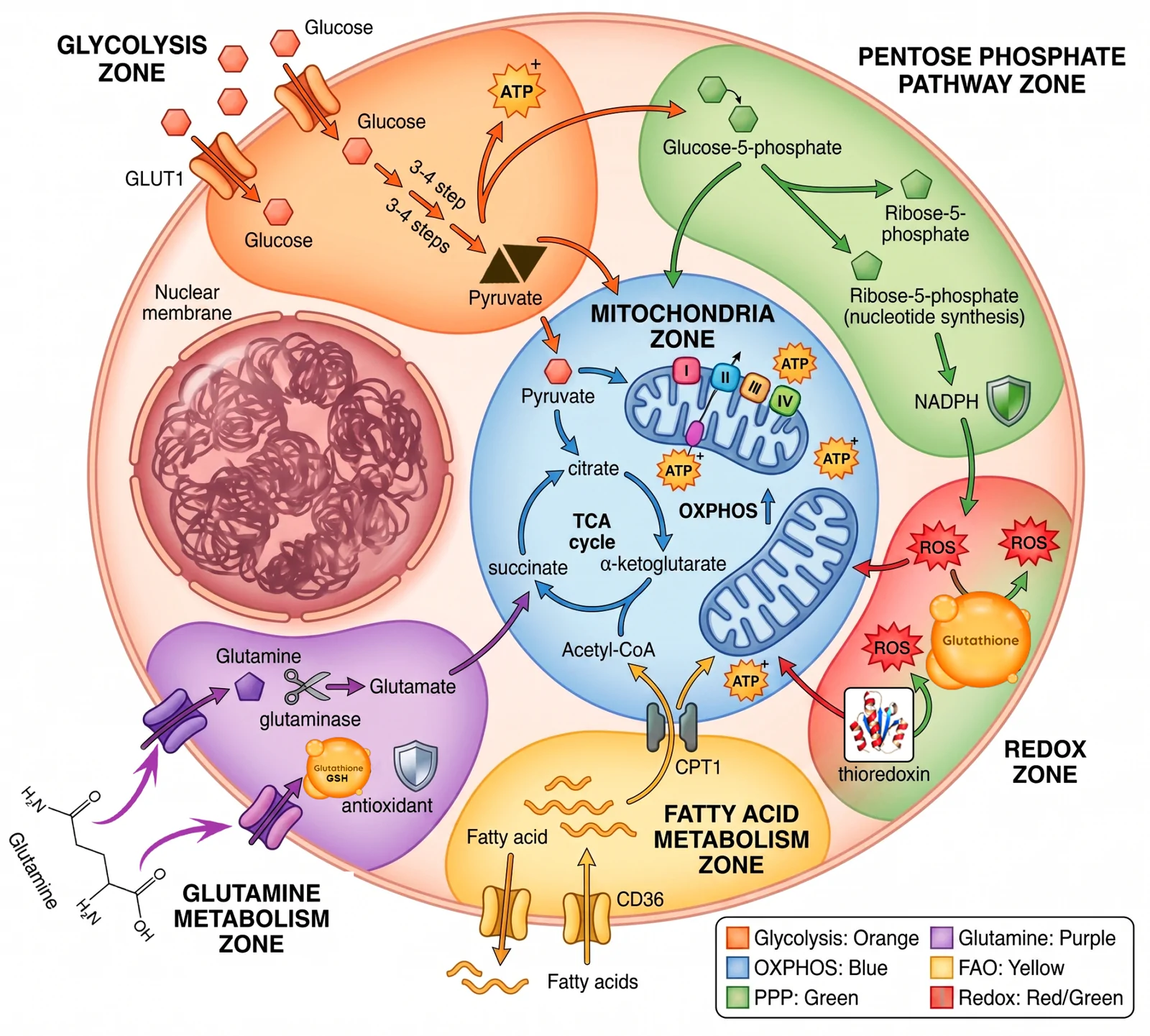
Metabolic reprogramming supports rapid proliferation in AML cells. Schematic of the principal metabolic programs reprogrammed in AML, organized by functional zone: glycolysis (orange), the pentose phosphate pathway (green), mitochondrial OXPHOS and the TCA cycle (blue), glutamine metabolism (purple), fatty acid oxidation (yellow), and redox/antioxidant systems (red/green). Glucose entering through GLUT1 is converted to pyruvate and lactate or diverted into biosynthesis, while glutaminolysis and fatty acid oxidation feed the TCA cycle and OXPHOS, and NADPH/glutathione/thioredoxin systems buffer reactive oxygen species. AML: acute myeloid leukemia, OXPHOS: oxidative phosphorylation, TCA: tricarboxylic acid, GLUT1: Glucose Transporter 1, NADPH: Nicotinamide Adenine Dinucleotide Phosphate, CPT1: Carnitine Palmitoyltransferase 1, FAO: Fatty Acid Oxidation, PPP: Pentose Phosphate Pathway. The figure was created with the AI Integrated CANVA-Pro online tool.

Before considering individual pathways, it is helpful to summarize the core metabolic alterations that define AML and to indicate, for each, how it differs from the generic reprogramming seen across cancers [10]. To sustain uncontrolled proliferation, leukemic cells rewire their metabolism: they absorb large amounts of glucose and glutamine and convert them into energy and biosynthetic building blocks even when oxygen is present (the Warburg effect), they redirect carbon into the pentose phosphate pathway and into lipid synthesis, and they remodel redox metabolism to buffer reactive oxygen species (ROS). As shown in Figure 1, the principal programs are aerobic glycolysis, oxidative phosphorylation (OXPHOS) fueled by glutaminolysis and fatty acid oxidation (FAO), the pentose phosphate pathway (PPP), and the thioredoxin/glutathione redox systems.

Crucially, none of these programs is exclusive to AML; each operates in other malignancies and in activated normal cells. The features that are comparatively distinctive in AML and that are emphasized in the subsections below include the marked dependence of leukemic stem cells (LSCs) on OXPHOS, the systemic induction of insulin resistance by leukemic blasts, and the strong association of specific driver mutations (*FLT3-ITD*, *IDH1/2*, *TET2*, *RUNX1*) with particular metabolic dependencies [10].

### 2.1. Glucose Metabolism and the Warburg Effect in AML

Although heightened glycolytic flux is a well-established hallmark of cancer and leukemogenesis [11,12], the extent to which leukemic cells engage in aerobic glycolysis (the Warburg effect) varies across hematological malignancies, directly influencing tumor aggressiveness and therapeutic response. AML blasts typically display marked glycolytic dependence to fuel rapid proliferation, providing essential precursor metabolites for nucleotide, amino acid, and lipid biosynthesis [13]. Parallel flux through the oxidative PPP further generates NADPH to maintain redox homeostasis while supplying ribose-5-phosphate for nucleic acid assembly [14]. Notably, glycolytic activity in AML is dynamically regulated by chromatin state; preclinically, histone deacetylase inhibitors (HDACi) have been shown to suppress specific glycolytic gene transcripts, illustrating functional crosstalk between epigenetic machinery and metabolic reprogramming [15]. Importantly, this metabolic reliance is non-uniform across the disease spectrum. Elevated glycolytic activity paradoxically correlates with favorable molecular subgroups, particularly core-binding factor (CBF) AML [16], reflecting metabolic heterogeneity that shapes sensitivity to targeted agents [12,17]. Collectively, these observations indicate that the magnitude of the Warburg phenotype rather than its simple presence serves as a critical prognostic indicator with direct implications for biomarker-driven patient stratification and glycolysis-targeted therapies [17].

### 2.2. Oxidative Phosphorylation

Despite the paradigm of metabolic reprogramming favoring aerobic glycolysis, OXPHOS remains an active contributor to ATP generation in primary AML cells through glutaminolysis and FAO [18]. Mitochondrial activity plays a permissive bioenergetic role during leukemogenesis and is essential for maintaining the metabolic demands of established leukemic populations [19]. AML myeloblasts frequently display enhanced mitochondrial mass and membrane potential, rendering them particularly dependent on OXPHOS for survival. High mitochondrial DNA content, commonly found in this malignancy, identifies OXPHOS-driven AML and represents a therapeutic vulnerability [20]. This OXPHOS dependence is one of the metabolic features that is comparatively distinctive in AML relative to many solid tumors: it is especially pronounced as LSCs, which rely on OXPHOS for self-renewal and chemoresistance [21], even though aerobic glycolysis dominates in the AML blast population. In vitro inhibition of Complex I with biguanides such as metformin impairs AML cell proliferation at millimolar concentrations, providing proof-of-concept for targeting mitochondrial respiration, though clinical translation requires more potent or combination approaches [22]. Consistent with this, when glucose uptake is blocked, AML cells adapt by converging upon mitochondrial respiration via enhanced Complex I function, creating an opportunity for combination strategies [23]. Many proliferating AML blasts exhibit substantial glycolytic activity [24], whereas relatively quiescent, ROS-low LSCs are metabolically less flexible and depend more strongly on OXPHOS for survival [25]. In newly diagnosed AML, LSC OXPHOS can be sustained by amino-acid catabolism [26,27]. By contrast, LSCs in relapsed disease or those resistant to venetoclax–azacitidine may use fatty-acid oxidation as an alternative source of OXPHOS fuel [28]. Therefore, reduced bulk blast viability should not be considered evidence of LSC eradication unless the primitive compartment is assessed directly, ideally using functional leukemia-initiating-cell assays [29,30].

### 2.3. Glutamine Metabolism

The synthesis, transport, and breakdown of the amino acid glutamine is highly altered in AML cells, which exhibit a pronounced reliance on glutamine to sustain critical metabolic functions. Upon cellular uptake, glutamine is metabolized to glutamate and subsequently converted to α-ketoglutarate through the action of glutaminase enzymes [10]. AML cells predominantly utilize reductive glutamine metabolism, which generates NADPH via malic enzyme and maintains redox homeostasis through reduced glutathione production [31]. Metabolomic analyses of patient samples have revealed approximately 7.5-fold higher α-ketoglutarate levels in AML cells compared to normal peripheral blood mononuclear cells, indicating markedly enhanced glutaminolysis [32]. AML cell lines depleted of glutamine-derived metabolites exhibit decreased nucleotide pools, accumulation of the oncometabolite 2-hydroxyglutarate (in IDH-mutant cases), and altered expression of pathway enzymes [33]. Leukemic cells persisting during FLT3 tyrosine kinase inhibitor treatment critically depend on maintaining mitochondrial metabolism through glutamine oxidation [34], and TET2-mutant leukemia exhibits a unique metabolic signature with heightened glutamine addiction, rendering these cells particularly vulnerable to glutaminase inhibition [35].

### 2.4. Fatty Acid Metabolism

Altered lipid metabolism represents another hallmark of metabolic reprogramming in cancer. Increased flux of glucose-derived carbon into lipids supports membrane biosynthesis for rapidly proliferating cells [35], and FAO provides an alternative source of acetyl-CoA for the TCA cycle, particularly under conditions of glucose or glutamine deprivation [36]. AML cells, including LSCs, depend on FAO for survival and chemoresistance. Inhibition of carnitine palmitoyltransferase 1 (CPT1), the rate limiting enzyme for mitochondrial fatty acid import, preferentially impairs AML cell and LSCs viability in preclinical models relative to normal hematopoietic progenitors [37]. Metabolic reprogramming in AML is compartment-specific: LSCs depend strongly on OXPHOS, and in relapsed disease or during resistance to venetoclax–azacitidine they can increase FAO as an alternative source of oxidative fuel. This adaptation links fatty-acid metabolism to LSC persistence and treatment resistance [28,37].

### 2.5. Redox Metabolism

Reactive oxygen species play a paradoxical role in cancer, acting as signaling molecules that promote proliferation while also triggering cell death through apoptosis and DNA damage when present at excessive levels [38]. In AML specifically, NADPH oxidase (NOX)-derived ROS are overproduced and promote leukemic proliferation, and AML cells adapt to this oxidative burden by upregulating antioxidant defense mechanisms, including the pentose phosphate pathway (NADPH generation), glutathione synthesis, and thioredoxin systems [39,40]. NADPH production through glucose-6-phosphate dehydrogenase-driven glycolysis and the transaldolase-dependent pathway enables ROS detoxification while supporting anti-apoptotic BCL-2 function [41]. In contrast, normal myeloid progenitors exhibit a more limited capacity for cytoprotection, suggesting a therapeutic window for redox-modulating agents [41]. Combinations of thioredoxin reductase inhibitors with pro-oxidant agents demonstrate synergistic toxicity against AML cells through profound ROS generation and mitochondrial disruption [40,42].

## 3. Inhibitors Targeting Metabolic Pathways as Potential Drugs to Treat AML

**Figure 2:**
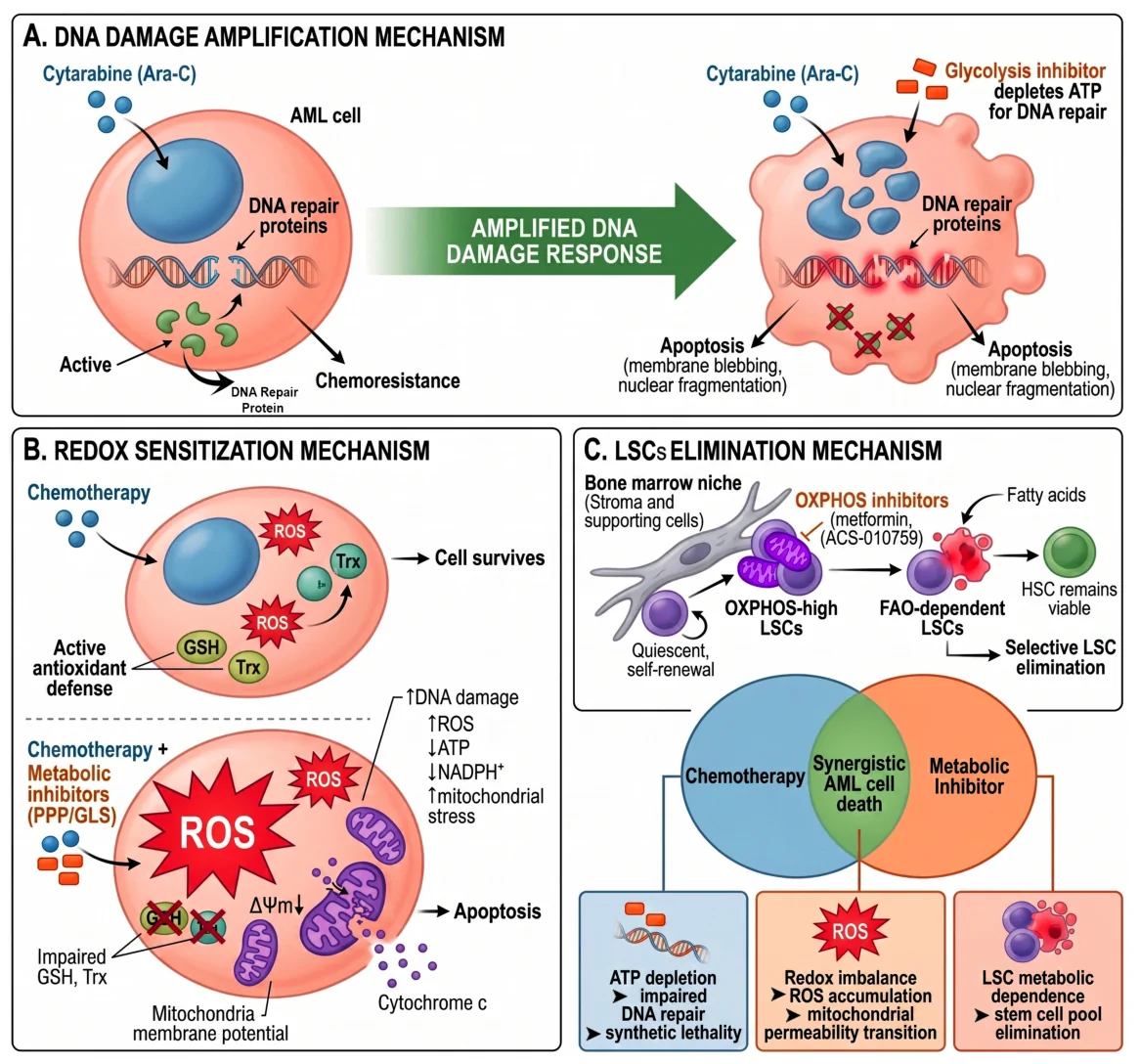
Mechanisms of synergy between metabolic inhibitors and chemotherapy in AML. (**A**) DNA-damage amplification: glycolysis inhibitors deplete the ATP required for DNA repair, converting chemoresistance into amplified DNA damage and subsequent apoptosis. (**B**) Redox sensitization: by impairing glutathione and thioredoxin antioxidant defenses, metabolic inhibitors allow chemotherapy-induced ROS to accumulate, triggering mitochondrial permeabilization. (**C**) LSCs elimination: OXPHOS inhibitors target the OXPHOS- and FAO-dependent LSCs compartment while sparing normal hematopoietic stem cells. The Venn diagram summarizes the convergence of chemotherapy and metabolic inhibition on synergistic AML cell death. AML, acute myeloid leukemia; Ara-C, cytarabine; ATP, adenosine triphosphate; DNA, deoxyribonucleic acid; FAO, fatty-acid oxidation; GLS, glutaminase; GSH, reduced glutathione; HSC, hematopoietic stem cell; LSC, leukemic stem cell; NADPH, reduced nicotinamide adenine dinucleotide phosphate; OXPHOS, oxidative phosphorylation; PPP, pentose phosphate pathway; ROS, reactive oxygen species; Trx, thioredoxin; ΔΨm, mitochondrial membrane potential; ↓, mean decrease; ↑, mean elevation or increase. The figure was created by using AI Integrated Canva-Pro online tool.

**Figure 3:**
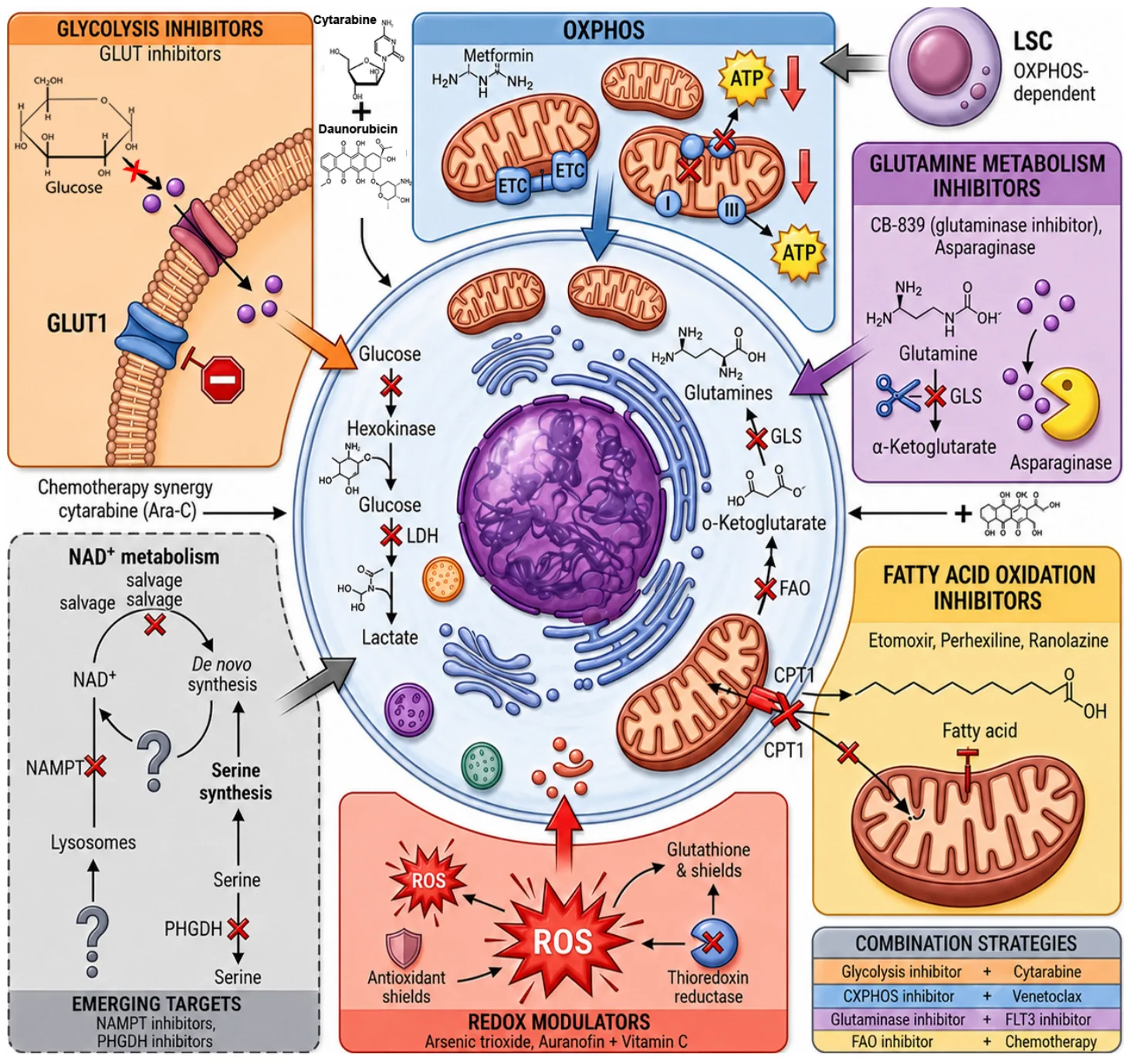
Therapeutic targeting of acute myeloid leukemia metabolism: metabolic inhibitors in clinical development for combination therapy. Schematic mapping drug classes onto their metabolic targets: glycolysis inhibitors (2-DG, MCT1 inhibitors, GLUT inhibitors), OXPHOS inhibitors (metformin, IACS-010759, atovaquone), glutamine-metabolism inhibitors (CB-839, asparaginase), fatty-acid-oxidation inhibitors (etomoxir, perhexiline, ranolazine), redox modulators (arsenic trioxide, auranofin plus vitamin C), and emerging targets (NAMPT and PHGDH inhibitors). The combination-strategies panel pairs each inhibitor class with its principal chemotherapeutic or targeted partner. 2-DG, 2-deoxy-D-glucose; Ara-C, cytarabine (cytosine arabinoside); ATP, adenosine triphosphate; CPT1, carnitine palmitoyltransferase 1; ETC, electron transport chain; FAO, fatty acid oxidation; *FLT3*, FMS-like tyrosine kinase 3; *GLS*, glutaminase; GLU, glucose/glutamate; GLUT/GLUT1, glucose transporter/glucose transporter 1; LDH, lactate dehydrogenase; LSC, leukemia stem cell; *MCT1*, monocarboxylate transporter 1; NAD^+^, nicotinamide adenine dinucleotide (oxidized); *NAMPT*, nicotinamide phosphoribosyltransferase; OXPHOS, oxidative phosphorylation; *PHGDH*, phosphoglycerate dehydrogenase; ROS, reactive oxygen species. The figure was created with AI Integrated CANVA-Pro online tool.

**Figure 4:**
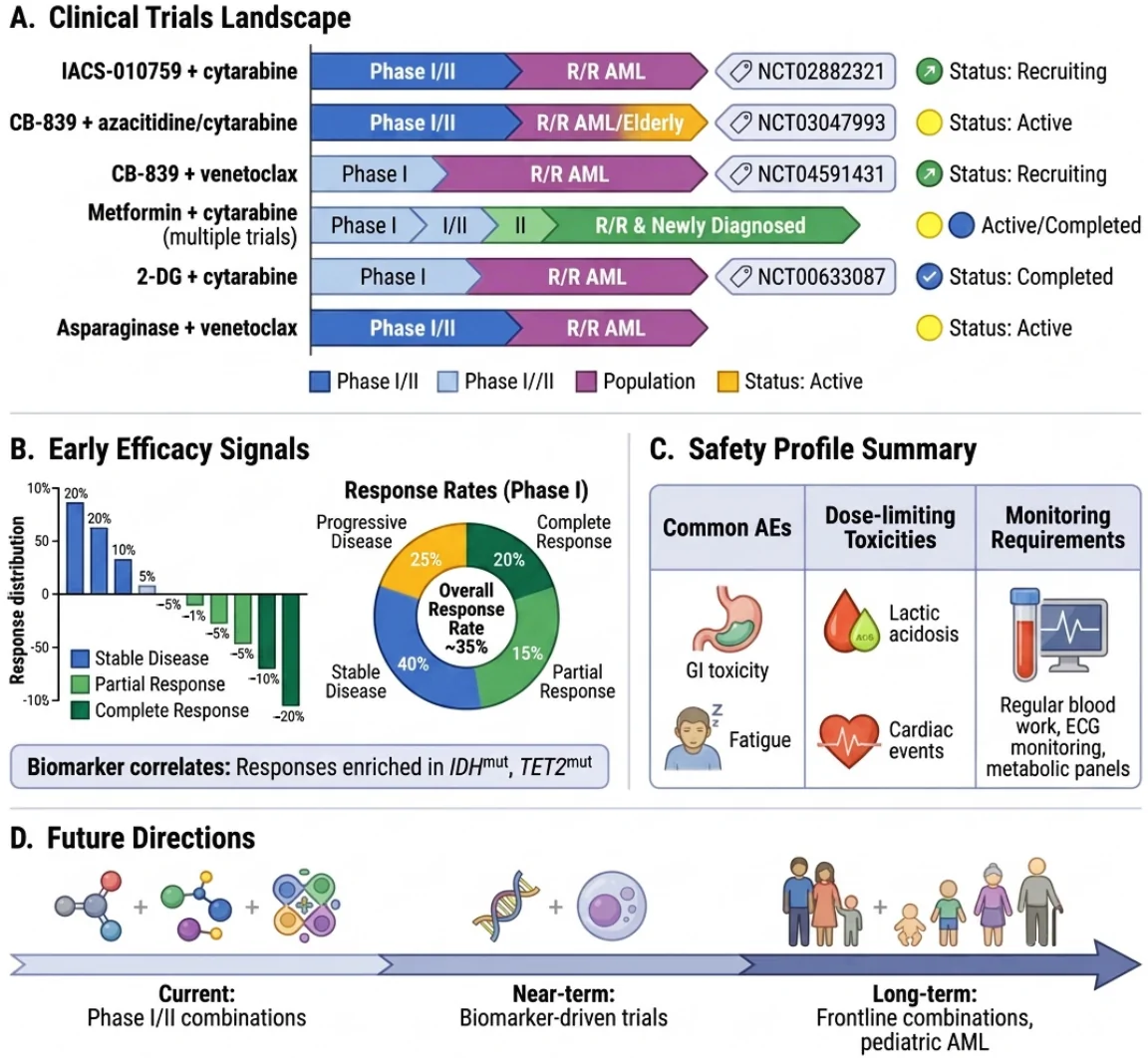
Clinical pipeline of metabolic modulators in AML. (**A**) Clinical-trials landscape, showing representative metabolic-inhibitor–chemotherapy combinations, their trial phase, target population, registry identifier, and recruitment status. (**B**) Early efficacy signals, with an illustrative response distribution and an approximate overall response rate of ∼35% in phase I combinations, enriched in IDH- and TET2-mutant cases. (**C**) Safety-profile summary of common adverse events, dose-limiting toxicities, and monitoring requirements. (**D**) Future directions, from current phase I/II combinations toward biomarker-driven and frontline/pediatric studies. 2-DG, 2-deoxy-D-glucose; AEs, adverse events; AML, acute myeloid leukemia; CR, complete response; EKG, electrocardiogram; GI, gastrointestinal; *IDH*^mut^, isocitrate dehydrogenase (mutated); ECG, electrocardiogram; NCT, National Clinical Trial (ClinicalTrials.gov identifier); PD, progressive disease; PR, partial response; R/R AML, relapsed or refractory acute myeloid leukemia; SD, stable disease; *TET2*^mut^, tet methylcytosine dioxygenase 2 (mutated). The figure was created with AI Integrated CANVA-Pro online tool.

Although our biological understanding of AML has grown significantly, treatments are still evolving to fully translate these insights into universal cures. In the effort to develop new treatments for AML, the synergy between inhibitors of the biological pathways discussed below and standard chemotherapy is being explored (Figure 2). Currently, many of these drugs are in clinical trials. Those that have reached phase III are, in principle, ready to inform AML treatment and are flagged as such (Figure 3, Table 1), while the broader clinical pipeline is described in Figure 4.

### 3.1. Mechanisms of Synergy: DNA Damage Amplification, Redox Sensitization, Mitochondrial Priming and Drug Resistance Reversal

Prior to examining specific pharmacological agents, it is essential to establish the mechanistic rationale underlying synergistic rather than merely additive interactions between metabolic modulators and conventional chemotherapy. The extensive metabolic reprogramming of AML generates distinct metabolic dependencies that differentiate leukemic blasts from normal hematopoietic stem and progenitor cells (HSPCs) [10]. By inhibiting pathways vital for AML survival, metabolic modulators induce bioenergetic crisis and oxidative stress, deplete intracellular ATP and NADPH pools, and restrict key metabolites required for DNA repair following chemotherapy-induced damage (Figure 2) [58]. This synergistic cytotoxicity is driven by four primary mechanisms: amplification of the DNA damage response, redox sensitization, mitochondrial priming, and the reversal of therapeutic resistance, including the eradication of LSCs.

#### 3.1.1. Amplification of the DNA Damage Response

Glycolysis inhibitors activate a cell-intrinsic metabolic checkpoint that suppresses DNA-damage response pathways, inducing immediate apoptosis upon subsequent exposure to cytotoxic agents [59]; OXPHOS inhibition similarly aggravates DNA damage and enhances apoptosis downstream of chemotherapy [60].

#### 3.1.2. Redox Sensitization

Many anticancer agents, including cytarabine and anthracyclines, act partly through ROS generation. By limiting NADPH production via the pentose phosphate pathway or glutaminolysis, metabolic inhibitors impair ROS detoxification and sensitize AML cells to chemotherapy-induced oxidative stress [38].

#### 3.1.3. Mitochondrial Priming

Targeting mitochondrial structure or transcription sensitizes AML cells to BCL-2 inhibition by suppressing OXPHOS and enhancing apoptotic priming; combinations of mitochondrial inhibitors with venetoclax synergistically induce apoptosis and loss of mitochondrial membrane potential [61].

#### 3.1.4. Overcoming Resistance and Targeting LSCs

Novel metabolic agents can resensitize venetoclax-resistant AML cells through induction of mitochondrial stress, targeting the LSCs responsible for relapse [62].

### 3.2. Glycolysis Inhibitors

Compounds or drugs targeting glycolytic enzymes or glucose transport block the cellular pathway that breaks down glucose for energy to restrict energy production, which could synergize with conventional chemotherapy (Figure 3) [63].

#### 3.2.1. 2-Deoxyglucose (2-DG)

This glucose analog inhibits hexokinase, the first committed step of glycolysis, and activates a metabolic checkpoint that suppresses the DNA-damage response, inducing apoptosis upon subsequent cytotoxic exposure [64,65]. Preclinically, 2-Deoxyglucose (2-DG) synergizes with cytarabine across U937, HL-60, and KG-1a cells, and partially restores chemosensitivity in resistant lines cytarabine IC50 850 to 120 nM) through AMPK activation, impaired DNA repair, and dNTP depletion [43]. Clinically, a phase I/II trial of 2-DG with cytarabine in relapsed/refractory AML (NCT00633087) reported acceptable safety and preliminary anti-leukemic activity; this combination remains early-phase.

#### 3.2.2. Glucose-Transport Inhibitors

Potent small-molecule inhibitors of Class I glucose transporters (e.g., the GLUT1 inhibitor BAY-876) block glucose uptake and glycolysis, producing a compensatory increase in mitochondrial respiration that can be therapeutically exploited [66]. In NRAS-mutant cell lines (OCI-AML3, THP-1), BAY-876 plus cytarabine is synergistic, and adding metformin further deepens synergy by blocking the compensatory OXPHOS shift [44,67]. These agents remain preclinical.

#### 3.2.3. MCT1 Inhibitors

Selective inhibitors of monocarboxylate transporter 1 (MCT1; e.g., AZD3965) block lactate export, disrupt glycolytic flux and intracellular pH, and cooperate with DNA-damaging agents [68]. AZD3965 with cytarabine is synergistic in glycolytic lines (MOLM-13, MV4-11) while sparing normal CD34+ cells [45], and a phase I study of AZD3965 drug in advanced cancer established a recommended dose for MCT1-high/MCT4-low tumors [69].

### 3.3. Oxidative Phosphorylation Inhibitors

Given the marked dependence of AML, particularly the LSC compartment, on OXPHOS, mitochondrial inhibitors such as metformin, IACS-010759, inhibitors of mitochondrial transcription, and atovaquone have emerged as promising agents in AML management [70].

#### 3.3.1. Metformin

This widely used antidiabetic inhibits Complex I, reduces ATP production, and activates AMPK signaling, showing broad anti-leukemic activity and synergy with several chemotherapeutics [71,72]. The strongest preclinical synergy is with idarubicin and is most pronounced in cells with high basal OXPHOS, where metformin lowers mitochondrial cell membrane potential, increases anthracycline accumulation, and resensitizes cytarabine-resistant lines [46]. In current practice, metformin is not an approved anti-leukemic drug; as monotherapy it showed minimal single-agent activity in relapsed/refractory AML [73], and it is now pursued almost exclusively as a combination partner. Metformin-treated cells can adapt by increasing glycolysis and NAD+ production, a resistance mechanism that can be bypassed by co-targeting nicotinamide phosphoribosyltransferase (NAMPT) [20,74,75]. Multiple early-phase trials are evaluating metformin with cytarabine, venetoclax, or azacitidine [76].

#### 3.3.2. IACS-010759

This potent, selective Complex I inhibitor reduces ATP, activates AMPK, and increases mitochondrial stress, potentiating targeted agents and chemotherapy [77]. It synergizes with cytarabine most strongly in FLT3-ITD lines- and with venetoclax by triggering BIM dependent apoptosis (encoded by the *BCL2L11* gene) in a TP53 dependent manner [47]. A first in human phase I program in advanced solid tumors and AML defined its tolerability and pharmacodynamics [77], and a phase I trial in AML (NCT02882321) is establishing the recommended phase II dose [78].

#### 3.3.3. Inhibitors of Mitochondrial Transcription

First-in-class inhibitors of mitochondrial RNA polymerase (POLRMT; e.g., IMT1) reduce mitochondrial-DNA transcription and impair OXPHOS biogenesis [79]. IMT1 with venetoclax is synergistic, including in venetoclax-resistant lines and refractory primary samples, acting through the integrated stress response and BAK-dependent apoptosis while sparing normal progenitors [49,80].

#### 3.3.4. Atovaquone

Originally an anti-malarial, atovaquone inhibits Complex III and shows repurposing synergy with cytarabine, with 85–95% reduction in CD34+CD38− LSCs populations in primary samples; the effect depends strictly on Complex III activity and on aspartate depletion [81,82,83]. It remains preclinical/early-phase in AML.

### 3.4. Inhibitors of Glutamine Metabolism

Inhibitors of glutamine metabolism block the pathways tumor cells use to metabolize glutamine, depriving them of anaplerotic carbon, reducing equivalents, and nucleotide precursors [31]. In the studies below, the “conventional agents” combined with these inhibitors are the standard cytotoxics cytarabine and the anthracyclines (daunorubicin, idarubicin), whereas the “targeted agents” are venetoclax (a BCL-2 inhibitor), the FLT3 inhibitor quizartinib, and IDH inhibitors.

#### 3.4.1. Glutaminase Inhibitors

CB-839 (telaglenastat) blocks the conversion of glutamine to glutamate via glutaminase 1 (GLS1) and synergizes with cytarabine and venetoclax, most strongly in IDH1/2-mutant and TET2-mutant cell lines, by depleting glutamate, glutathione, and aspartate and impairing the DNA-damage response [50,51,84,85]. CB-839 is in clinical evaluation with azacitidine or cytarabine (NCT03047993) and with venetoclax in IDH-mutant AML (NCT04591431) [86].

#### 3.4.2. Asparaginase Formulations

Long-acting crisantaspase (pegcrisantaspase) depletes plasma glutamine and asparagine, creating metabolic stress. With venetoclax it is synergistic, particularly in RUNX1-mutant lines, via GCN2–ATF4–CHOP activation and BAK-dependent apoptosis [42,87]; a phase I study of pegcrisantaspase plus venetoclax in relapsed/refractory AML showed acceptable safety and promising activity in molecularly defined subsets [88].

#### 3.4.3. Combination with FLT3 Inhibitors

Quizartinib-resistant FLT3-ITD cells acquire enhanced glutamine dependence; CB-839 plus quizartinib eliminates resistant clones through combined blockade of FLT3 signaling and glutamine-fueled mitochondrial metabolism [34,89].

### 3.5. Inhibitors of Fatty Acid Oxidation

FAO inhibitors aim to eliminate LSCs and overcome chemoresistance by blocking the breakdown of lipids that leukemia cells rely on for energy, thereby shifting cellular metabolism away from FAO and toward glucose oxidation [90]. In this regard, the FAO inhibitor avocatin B an odd-numbered carbon lipid derived from avocado fruit induces selective apoptosis and growth inhibition in AML cells. Furthermore, combining avocatin B with the conventional anti-AML agent cytarabine markedly increases intracellular ROS and exerts highly synergistic antineoplastic effects [91].

#### 3.5.1. CPT1 Inhibitors

Etomoxir and perhexiline block mitochondrial fatty-acid import (the rate-limiting FAO step), selectively impairing AML proliferation while sparing normal progenitors [37]. Etomoxir with cytarabine or venetoclax is synergistic and selectively depletes LSCs (90–95% versus 50–70% for bulk cells), partly through GSK3β-mediated MCL-1 downregulation [28,53]; perhexiline with venetoclax overcomes MCL-1-driven venetoclax resistance [54].

#### 3.5.2. Ranolazine

This partial FAO inhibitor, approved for angina, shows preclinical activity and synergizes with BCL-2-family inhibitors through regulation of BAK-dependent mitochondrial permeability transition, with particular activity under bone-marrow adipose-niche conditions [53,55,92].

### 3.6. Redox Modulators

Redox modulators are agents that deliberately perturb the cellular oxidant–antioxidant balance either by generating ROS (pro-oxidants) or by disabling antioxidant defenses such as the thioredoxin and glutathione systems to push the already elevated ROS of AML cells beyond the threshold compatible with survival [39]. They exploit the elevated ROS levels and enhanced antioxidant capacity of AML cells.

#### 3.6.1. Thioredoxin Reductase Inhibitors Plus Vitamin C

Auranofin (a thioredoxin reductase inhibitor) combined with high-dose vitamin C (a pro-oxidant) produces synergistic toxicity, most strongly in TP53-mutant and FLT3-ITD lines, through a 5–8-fold ROS rise, glutathione/NADPH depletion, and ferroptotic as well as apoptotic death, with relative sparing of normal stromal cells [40,41,42,56,93].

#### 3.6.2. Arsenic Trioxide

Arsenic trioxide induces oxidative stress through glutathione depletion and thioredoxin reductase inhibition; it is standard therapy for acute promyelocytic leukemia (APL) and shows activity in non-APL AML in combination, for example with the glutathione-synthesis inhibitor buthionine sulfoximine [86,94].

### 3.7. Emerging Metabolic Targets

The metabolic plasticity of AML creates additional, highly specific vulnerabilities. Serine and one-carbon metabolism is upregulated to support nucleotide synthesis and methylation, and inhibitors of phosphoglycerate dehydrogenase (PHGDH), the rate-limiting serine-synthesis enzyme, are in preclinical development [87,95]. Because metformin-treated cells adapt by increasing NAD+ production through NAMPT, combining metformin with NAMPT inhibitors is a rational resistance-bypass strategy [74,75]. Finally, high mitochondrial DNA content identifies OXPHOS-driven AML and predicts sensitivity to mitochondrial inhibitors, enabling a more personalized approach [20,96].

### 3.8. Clinical Pipeline and Association with Outcome

The agents above have entered clinical evaluation chiefly in early-phase, combination settings; their trial status and the available outcome data are summarized here and in (Figure 4). Most of the ongoing studies focused metabolic-inhibitor combinations remain in phase I/II. Representative studies include 2-DG plus cytarabine (NCT00633087, completed/early-phase), IACS-010759 plus cytarabine (NCT02882321), metformin with cytarabine, venetoclax, or azacitidine, CB-839 with azacitidine/cytarabine (NCT03047993) or venetoclax in IDH-mutant AML (NCT04591431), and pegcrisantaspase plus venetoclax [76,78,88,97].

With respect to association with outcome, three patterns emerge from these early data. First, response rates: preliminary phase I data indicate that metabolic-inhibitor–chemotherapy combinations can induce responses in relapsed/refractory AML, including in previously treated patients, although complete-remission rates vary with population and regimen [98]. Second, duration of response: sustained responses have been observed, particularly in IDH-mutant AML treated with glutaminase-inhibitor–containing regimens, though relapse remains common [99]. Third, and most relevant to outcome, specific molecular contexts track with benefit: elevated baseline OXPHOS gene expression with response to metformin-containing regimens [100], high glutaminase expression and IDH1/2 or TET2 mutation with glutaminase-inhibitor sensitivity [101,102], and RUNX1 mutation with response to asparaginase-based combinations [97]. To date, no metabolic inhibitor has advanced to a registrational phase III AML combination, so none is yet “ready to use” outside trials; where phase III data exist for related antimitochondrial agents (e.g., CPI-613/devimistat) they have been negative, underscoring the gap between preclinical promise and clinical benefit.

Importantly, the strength of this evidence is uneven. For example, the reported activity of 2-DG plus cytarabine was evaluated only in KG1-a–derived cells, without normal CD34^+^ controls, primary patient samples, or in vivo validation [43]. The early clinical record also demonstrates that mechanistic plausibility is insufficient: encouraging phase I and II findings with devimistat [98,103] were not confirmed in the pivotal phase III ARMADA 2000 trial. This study enrolled patients aged ≥50 years with relapsed or refractory AML and randomized them to receive devimistat combined with high-dose cytarabine and mitoxantrone (CHAM) versus one of three standard salvage regimens. Among 200 randomized patients, the complete remission rate was 20.4% in the devimistat arm compared with 21.6% in the control arm, and median overall survival was 8.9 months versus 6.2 months. Thus, despite a consistent safety profile, the addition of devimistat did not improve remission or survival outcomes [104]. Similarly, IACS-010759 had a narrow therapeutic index because elevated blood lactate and neurotoxicity prevented sustained target exposure [77]. Collectively, these findings indicate that reproducibility, clinically relevant exposure, and a demonstrable therapeutic window are necessary before a metabolic combination can be considered clinically promising.

## 4. Multitherapy as a Promising Opportunity in AML

A recurring theme in AML therapy based on metabolic inhibitors is that none of them is curative, because AML cells almost always retain an alternative route to meet their energetic and biosynthetic needs. This makes rational multi-target (combination) therapy a distinct opportunity rather than merely an incremental improvement (Table 1).

The case for combinations depends first on the differential metabolic dependence of leukemic versus normal cells. Accumulating preclinical evidence indicates that targeting specific metabolic dependencies can differentially impair leukemic blasts and LSCs while demonstrating a potential therapeutic window relative to healthy HSPCs [37]; IMT1 plus venetoclax eliminates AML cells in co-culture while preserving normal colony-forming-unit potential, owing to the 3–5-fold higher basal mitochondrial-translation rate of AML cells [49]; and combined GLUT1/OXPHOS inhibition restrains the metabolic plasticity of primary AML, especially RUNX1-mutant cells, while sparing healthy cells. This selectivity arises because normal progenitors retain metabolic flexibility and adapt to inhibition of a single pathway, whereas AML cells are frequently “addicted” to a particular program [73].

The mechanistic basis for this selectivity can be made concrete with OXPHOS inhibition as an example. OXPHOS inhibition reduces ATP, activates AMPK signaling, and increases mitochondrial stress in AML cells while exerting minimal effects on normal progenitors [105]. In detail, AML cells particularly LSCs operate at near maximal respiratory capacity with minimal spare reserve. Consequently, Complex I blockade severely depletes their ATP supply and forces a glycolytic shift that creates a secondary metabolic vulnerability. By contrast, normal hematopoietic progenitors possess greater spare respiratory capacity and can better tolerate this metabolic perturbation. However, despite this proposed therapeutic index in preclinical models, the clinical phase I evaluation of IACS-010759 demonstrated that dose-limiting toxicities, specifically elevated blood lactate and neurotoxicity, prevented sustained target inhibition, thereby narrowing rather than widening the therapeutic window [77,105].

Building on this, rationally designed combinations exploit the predictable adaptive responses described earlier. Glucose-transport blockade drives a compensatory rise in OXPHOS that is neutralized by adding metformin [44]; glutaminase inhibition that depletes glutathione potentiates redox-active chemotherapy [106]; and FAO inhibition that lowering MCL-1 sensitizes AML cells to venetoclax [28]. Because these adaptations are sequential, the order in which agents are given matters: 2-DG and metformin produce the greatest synergy when administered before, rather than simultaneously with, cytarabine, so the schedule of administration not only the choice of partner must be optimized [43,107]. Triple combinations (e.g., GLUT1 inhibitor plus metformin plus cytarabine) further suppress the emergence of resistant clones in long-term culture [44].

Combinations are also a strategy against established resistance. During relapse, LSCs pretreated with venetoclax and azacitidine upregulate FAO to compensate for OXPHOS inhibition [28], and therapy-resistant cells can sustain the TCA cycle by importing lactate through MCT1; pharmacologically targeting these bypasses restores sensitivity to BCL-2 or Bromodomain and Extra Terminal (BET) inhibition [28]. Collectively, these data position metabolism-directed multitherapy as a means of targeting metabolically heterogeneous, relapse-driving populations that single agents cannot eradicate [108].

## 5. Challenges and Future Directions

Three categories of problem must be solved before metabolism-directed combinations can change AML practice: they must be safe and tolerable when added to cytotoxic therapy; they must contend with the metabolic heterogeneity and plasticity of the disease; and they must be tested in trials whose designs include biomarker guided patient selection and can demonstrate clinical benefit.

### 5.1. Safety, Toxicity, and Tolerability

Metabolic inhibitors have generally shown favorable safety when combined with standard treatment, with dose-limiting toxicities that are usually manageable and consistent with each agent’s known profile [78]. The principal toxicities are gastrointestinal (nausea, vomiting, diarrhea), which may limit single-agent dose escalation but are manageable with supportive care and schedule optimization [109,110]; and metabolic disturbances, notably lactic acidosis with glycolysis inhibitors and hypoglycemia with agents that impair glucose utilization, both requiring monitoring of acid–base and glucose status. Optimizing safety in combination regimens therefore depends on careful scheduling, patient selection, and supportive care [111].

### 5.2. Metabolic Heterogeneity and Plasticity

Metabolic dependencies in AML exhibit marked interpatient and intratumoral heterogeneity. Under pharmacological selective pressure, leukemic clones demonstrate substantial metabolic plasticity, rapidly upregulating compensatory pathways to bypass single-agent inhibition and drive acquired resistance [58,112]. This intrinsic adaptability is further compounded by the bone marrow microenvironment, which confers protective bioenergetic support through intercellular mitochondrial transfer and lactate shuttling [113]. Together, cell-intrinsic plasticity and niche-mediated protection represent the primary biological barrier to durable metabolic targeting, underscoring the absolute necessity of the multi-targeted combination and rational scheduling frameworks detailed in Section 4.

From a clinical and global health perspective, resolving this metabolic heterogeneity introduces significant practical challenges. Precise characterization of metabolic and immunophenotypic subpopulations relies heavily on advanced platforms such as next generation sequencing of leukemia associated mutations and multiparameter flow cytometry that are not uniformly accessible worldwide. Because the mutational spectrum and metabolic behavior of AML vary geographically, biomarker-guided metabolic regimens risk remaining restricted to high-resource settings. Consequently, future efforts to stratify and treat AML must actively account for these global disparities in diagnostic access to avoid exacerbating inequities in precision oncology [113].

AML metabolism also shapes antileukemic immunity. AML blasts express and release arginase II, resulting in reduced arginine availability and suppression of T-cell proliferation [114]. Leukemia-derived lactic acid can further impair T-cell glycolysis, proliferation and effector function, whereas sodium-bicarbonate-mediated metabolic restoration enhanced the graft-versus-leukemia activity of murine and human donor T cells [115]. Because activated T and NK cells themselves depend on metabolic programs such as glucose uptake and glycolysis [116,117,118], metabolism-directed therapies could affect both malignant and immune-effector compartments. Such combinations should therefore be evaluated in immunocompetent or multicellular marrow models with separate leukemia-cell and immune-cell readouts.

### 5.3. Biomarker-Guided Patient Selection and Ongoing Trials

A consistent criticism of metabolic targeting reviews is that terms such as “biomarkers” and “stratification” are often invoked without clear operational definition. For a biomarker to be clinically useful, the biological feature being measured, the assay and specimen employed, and the treatment decision it is intended to inform must be specified. In a biomarker enriched trial, the assay result is used prospectively to select or stratify patients whose leukemia is predicted to depend on the targeted pathway. These biomarkers fall into two distinct categories, which should not be interchanged [119].

### 5.4. Genetic Biomarkers (Predictive of Drug Response)

These are recurrent driver mutations that, on the preclinical and early-clinical evidence above, predict sensitivity to a specific agent: IDH1/2 mutation predicts response to glutaminase inhibitors (mutant IDH creates dependence on exogenous glutamine for α-ketoglutarate) [120]; TET2 mutation predicts heightened glutamine addiction and glutaminase-inhibitor sensitivity [35]; FLT3-ITD predicts enhanced glycolytic flux and sensitivity to glycolysis inhibition and to FLT3-inhibitor combinations [50,121]; and TP53 status modifies response to mitochondrial-inhibitor and BCL-2-inhibitor combinations [122]. These are the biomarkers that can be acted upon today, because they are already measured at diagnosis.

### 5.5. Metabolic Measurements (Descriptive of Metabolic State, Not Synonyms of Response)

It is important to distinguish a metabolic biomarker from treatment response or chemosensitivity: a high glycolytic rate or a particular metabolite profile describes a cell’s metabolic state and is not, by itself, evidence of clinical benefit. Among these measurements, mitochondrial DNA content is the best-developed candidate predictive biomarker, as high mtDNA content identifies OXPHOS-driven AML and predicts sensitivity to mitochondrial inhibitors [20]. By contrast, comprehensive metabolite “signatures” derived from metabolomic profiling [123] are prognostic indicators and hypothesis-generating tools rather than validated, actionable biomarkers, and stable-isotope flux measurements of glycolytic and glutamine flux specifically can quantify pathway dependence in patient samples [124] but are not yet ready for routine clinical decision-making. The principal functional approaches are extracellular-flux analysis, which measures oxygen-consumption rate and extracellular acidification in viable patient-derived cells, and stable-isotope tracing coupled to mass spectrometry, which determines the contribution of a labeled substrate to downstream metabolites [125,126,127]. These measurements are influenced by specimen source, cellular composition and purity, viability, processing and culture conditions, substrate concentration, and normalization method [26,128,129]. Moreover, steady-state metabolite abundance is not equivalent to metabolic flux [128]. Accordingly, these assays require prospective standardization and clinical validation before they can be used as stand-alone tests for treatment selection.

On this basis, the reality is that of the candidate markers reviewed, only the recurrent driver mutations (IDH1/2, TET2, FLT3-ITD, TP53) are mature enough to stratify patients prospectively today; mitochondrial DNA content is the most promising metabolic candidate; and metabolite signatures and flux measurements require validation before they can guide therapy. Future trials should therefore embed prospective, mutation-based stratification (e.g., glutaminase inhibitors in IDH/TET2-mutant disease), incorporate metabolic measurements as exploratory endpoints, and extend to combinations with immunotherapy and to pediatric AML, whose metabolic dependencies may differ from those of adults [119,130]. Advances in high-resolution and single-cell metabolomics will progressively sharpen this selection by delineating the genomic, transcriptomic, and metabolic landscape of AML and revealing new, modulatable targets [9].

## 6. Conclusions

Targeting cellular metabolism has emerged as an attractive strategy for expanding therapeutic options in AML. Genetic, epigenetic, and microenvironmental factors collectively alter the metabolic state of leukemic cells, creating dependencies that can be exploited to enhance conventional therapy. Across glycolysis, OXPHOS, glutamine metabolism, fatty acid oxidation, and redox balance, small-molecule inhibitors potentiate cytarabine, anthracyclines, and venetoclax in preclinical models through amplification of DNA damage, redox sensitization, mitochondrial priming, and selective elimination of leukemic stem cells. Following this argument about the hallmarks of cancer through the metabolic programs of AML and the inhibitors that target them, to multi-target therapy and its remaining challenges the central message is that benefit is most likely to come from rationally scheduled combinations matched to a patient’s molecular context, rather than from any single agent. Early phase trials indicate that such combinations are feasible and can induce responses in relapsed/refractory disease, but metabolic heterogeneity and plasticity, micro environmental protection, unequal access to diagnostic technologies, and the lack of validated predictive biomarkers remain formidable obstacles. Continued investigation of resistance mechanisms, optimization of combination schedules, and development of robust, prospectively validated biomarkers for patient selection will determine whether metabolic modulators ultimately improve outcomes for the many patients still facing this challenging disease.

## Figures and Tables

**Table 1:** Summary of key in vitro studies evaluating metabolic inhibitor combinations in AML.

Inhibitor (Target)	Combination Partner	Model Systems	Primary Findings & Mechanism
Glycolysis			
2-Deoxyglucose [43]	Cytarabine	U937, HL-60, KG-1a cell lines	Resensitizes resistant cells (CI 0.3–0.5); activates AMPK, impairs DNA repair and depletes dNTPs.
BAY-876 (GLUT1) [44]	Cytarabine ± metformin	NRAS-mutant cell lines (OCI-AML3, THP-1)	High synergy (triple combo CI 0.15); blocks glucose uptake (↓85%) and induces compensatory OXPHOS.
AZD3965 (MCT1) [45]	Cytarabine	MOLM-13, MV4-11	Synergistic cytotoxicity (CI 0.3–0.5); spares CD34^+^ cells; drives lactate accumulation and replication stress.
Oxidative Phosphorylation			
Metformin [46]	Idarubicin, cytarabine	MLL/AF9, cytarabine-resistant cell lines	Resensitizes resistant cells (CI 0.25–0.45); reduces $\Delta \Psi_{m}$ (↓45%) and ATP (↓60%); downregulates CDA.
IACS-010759 [47]	Cytarabine, venetoclax	FLT3-ITD, TP53 wild-type cell lines	Strong synergy (CI 0.1–0.4); depletes OCR (↓85%) and ATP (↓75%); induces BIM-mediated apoptosis.
Atovaquone [48]	Cytarabine	High-mitochondrial-mass cell lines, LSCs	Selective LSC elimination (↓85–95%); depletes aspartate (↓75%) and elevates succinate.
IMT1 (POLRMT) [49]	Venetoclax	Venetoclax-resistant, AML samples	Potent synergy (CI 0.1–0.3); suppresses mtDNA-encoded subunits (↓60–80%); triggers BAK-dependent death.
Glutamine Metabolism			
CB-839 (GLS1) [50,51]	Cytarabine, venetoclax	IDH1/2, TET2-mutant, quizartinib resistant cell lines	Marked synergy (CI 0.1–0.5); clears resistant clones via severe glutamate (↓80%), GSH (↓60%), and ATP depletion.
Pegylated crisantaspase [52]	Venetoclax	RUNX1-mutant cell lines (Kasumi-1, SKNO-1)	60–85% viability reduction (CI 0.3–0.5); >95% glutamine depletion; activates GCN2-ATF4-CHOP stress pathway.
Fatty Acid Oxidation			
Etomoxir (CPT1) [53]	Cytarabine, venetoclax	High FAO cell lines, LSC-enriched populations	Preferentially targets LSCs (↓90–95% vs. bulk ↓50–70%); blocks palmitate oxidation; lowers MCL-1 via GSK3β.
ST1326 (Teglicar; CPT1A) [54]	Venetoclax (ABT-199)	THP-1, HL-60, Kasumi-1, MV4-11 and OCI-AML2 cell lines; primary AML blasts	Synergistic growth inhibition and apoptosis (CI < 1) in AML cell lines and ABT-199-sensitive and -resistant primary samples; prevents ABT-199-induced MCL-1 upregulation through reduced p-GSK3β and p-ERK signalling.
Ranolazine [55]	Cytarabine	Adipose-niche-mimicking conditions	Overcomes microenvironment resistance (CI 0.4–0.6); reduces FAO (↓; 50–60%) and OCR (↓; 40%).
Redox Modulation			
Auranofin + Vitamin C [56]	—	TP53-mutant, FLT3-ITD cell lines, primary AML	Profound synergy (CI 0.1–0.3); triggers ferroptosis via 5–8× ROS spike and severe GSH/NADPH depletion.
Arsenic trioxide + BSO [57]	—	High glutathione cell lines, LSCs	Eliminates LSCs (↓85%; CI 0.3–0.5); inhibits thioredoxin reductase (TrxR ↓60%) and depletes GSH (↓80%).

AML, acute myeloid leukemia; AMPK, AMP-activated protein kinase; ATF4, activating transcription factor 4; ATP, adenosine triphosphate; BAK, Bcl-2 homologous antagonist/killer; BIM, Bcl-2-like protein 11; BSO, buthionine sulfoximine; CD34, cluster of differentiation 34; CDA, cytidine deaminase; CHOP, C/EBP homologous protein; CI, combination index (Chou-Talalay); CPT1, carnitine palmitoyltransferase 1; DNA, deoxyribonucleic acid; dNTPs, deoxynucleoside triphosphates; FAO, fatty acid oxidation; FLT3-ITD, FMS-like tyrosine kinase 3 internal tandem duplication; GCN2, general control nonderepressible 2; GLS1, glutaminase 1; GLUT1, glucose transporter 1; GSH, glutathione; GSK3β, glycogen synthase kinase 3 beta; IC_50_, half-maximal inhibitory concentration; IDH1/2, isocitrate dehydrogenase 1/2; LSC, leukemic stem cell; MCL-1, myeloid cell leukemia 1; MCT1, monocarboxylate transporter 1; mtDNA, mitochondrial DNA; NADPH, nicotinamide adenine dinucleotide phosphate; NRAS, neuroblastoma RAS viral oncogene homolog; OCR, oxygen consumption rate; OXPHOS, oxidative phosphorylation; POLRMT, mitochondrial RNA polymerase; ROS, reactive oxygen species; RUNX1, runt-related transcription factor 1; TET2, tet methylcytosine dioxygenase 2; TP53, tumor protein p53; TrxR, thioredoxin reductase; $\Delta \Psi_{m}$, mitochondrial membrane potential. ↓, decrease relative to the corresponding experimental control or baseline.

## Data Availability

No new datasets were generated or analyzed in this study. All information discussed in this review is derived from previously published articles cited in the reference list.

## Abbreviations

The following abbreviations are used in this manuscript:

2-DG	2-Deoxyglucose
AML	Acute Myeloid Leukemia
AMPK	AMP-Activated Protein Kinase
ATF4	Activating Transcription Factor 4
ATP	Adenosine Triphosphate
AZD3965	Monocarboxylate Transporter 1 Inhibitor
BAY-876	Glucose Transporter 1 Inhibitor
BCL-2	B-Cell Lymphoma 2
BIM	BCL-2 Interacting Mediator of Cell Death
BSO	Buthionine Sulfoximine
CB-839	Telaglenastat (Glutaminase Inhibitor)
CBF	Core-Binding Factor
CDA	Cytidine Deaminase
CHOP	C/EBP Homologous Protein
CI	Combination Index
CPI-613	Devimistat
CPT1	Carnitine Palmitoyltransferase 1
DNA	Deoxyribonucleic Acid
dNTP	Deoxyribonucleoside Triphosphate
FAO	Fatty Acid Oxidation
FLT3	FMS-Like Tyrosine Kinase 3
FLT3-ITD	FMS-Like Tyrosine Kinase 3 Internal Tandem Duplication
GCN2	General Control Nonderepressible 2 Kinase
GLS1	Glutaminase 1
GLUT1	Glucose Transporter 1
GSH	Glutathione
GSK3β	Glycogen Synthase Kinase 3 Beta
HSPCs	Hematopoietic Stem and Progenitor Cells
IC_50_	Half-Maximal Inhibitory Concentration
IDH	Isocitrate Dehydrogenase
IDH1/IDH2	Isocitrate Dehydrogenase 1/2
IMT1	Mitochondrial RNA Polymerase Inhibitor
LSC/LSCs	Leukemic Stem Cell(s)
MCL-1	Myeloid Cell Leukemia 1
MCT1/MCT4	Monocarboxylate Transporter 1/4
NADPH	Nicotinamide Adenine Dinucleotide Phosphate (Reduced Form)
NAMPT	Nicotinamide Phosphoribosyltransferase
NOX	NADPH Oxidase
NRAS	Neuroblastoma RAS Viral Oncogene Homolog
OCR	Oxygen Consumption Rate
OXPHOS	Oxidative Phosphorylation
PHGDH	Phosphoglycerate Dehydrogenase
POLRMT	Mitochondrial RNA Polymerase
ROS	Reactive Oxygen Species
RUNX1	Runt-Related Transcription Factor 1
TCA	Tricarboxylic Acid (Cycle)
TET2	Tet Methylcytosine Dioxygenase 2
TP53	Tumor Protein p53
TrxR	Thioredoxin Reductase
$\Delta \Psi_{m}$	Mitochondrial Membrane Potential
